# Leveraging Programmatic Collaboration for a Radiopharmaceutical Clinic

**DOI:** 10.3390/cancers16071396

**Published:** 2024-04-02

**Authors:** Charles A. Kunos, Molly E. Martin, Michalis F. Georgiou, Russ A. Kuker, Aman Chauhan

**Affiliations:** 1 Department of Radiation Oncology, Sylvester Comprehensive Cancer Center, 1475 NW 12th Avenue, Suite 1500, Miami, FL 33136, USA; 2 Department of Radiology, Division of Nuclear Medicine, University of Iowa Health Care, Iowa City, IA 52242, USA; molly-martin@uiowa.edu; 3 Department of Radiology, Division of Nuclear Medicine, University of Miami, Miami, FL 33136, USA; 4 Department of Medicine, Division of Medical Oncology, University of Miami, Miami, FL 33136, USA; axc3268@med.miami.edu

**Keywords:** radiopharmaceutical, radionuclide treatment, theranostic, theranostic center, floor plan

## Abstract

**Simple Summary:**

Until recently, radiopharmaceuticals have been underutilized for the curative or the palliative treatment of cancer. Advances in a development program with investigational agents that have a radioactive payload as part of the studied drug product necessitate new ways of drug handling and administration as well as novel logistics for a safe and satisfactory patient experience. This review offers a roadmap for the clinical start-up and implementation of an integrated and leveraged programmatic collaboration for an academic theranostic center. Within this roadmap, there are complex issues of logistics, coordination, medical considerations, radioprotection, receipt and waste of radioactive agents, and patient release priorities. Regulatory agency input also adds to the layout dynamics of the theranostic center, but this roadmap does not cover specific requirements as United States state laws differ considerably.

**Abstract:**

Radiation oncologists, radiopharmacists, nuclear medicine physicians, and medical oncologists have seen a renewed clinical interest in radiopharmaceuticals for the curative or the palliative treatment of cancer. To allow for the discovery and the clinical advancement of targeted radiopharmaceuticals, these stakeholders have reformed their trial efforts and remodeled their facilities to accommodate the obligations of a program centered upon radioactive investigational drug products. Now considered informally as drugs and not beam radiotherapy, radiopharmaceuticals can be more easily studied in the traditional clinical trial enterprise ranging from phase 0–I to phase III studies. Resources and physical facilities allocated to radiopharmaceuticals have brought forth new logistics and patient experience for safe and satisfactory drug delivery. The clinical use of theranostic agents—that is, diagnostic and therapeutic radionuclide pairs—has accelerated radiopharmaceutical development.

## 1. Introduction

As the use of molecularly targeted agents, which are expected to increase tumor response and progression-free survival, evolves in oncology, there has been a corresponding rise in the development and recommendation for radiopharmaceuticals. A fundamental thought paradigm shift occurred in radiopharmaceutical development when these agents became considered effectual therapeutic drugs rather than just diagnostic medical imaging products [1]. Radiopharmaceuticals are accountable for long-lasting complete remissions in some cancers, like somatostatin receptor-positive gastroenteropancreatic neuroendocrine tumors, or for the durable palliation of others, like bone-metastatic prostate cancer [2,3,4]. The National Cancer Institute’s (NCI) backing of discovery-phase preclinical radiobiology and toxicology as well as support for phase 0–I to phase III development-phase clinical trials have moved molecularly targeted radioactive agents to the forefront of a national cancer drug research program [5]. 

For therapy, a radiopharmaceutical agent should demonstrate selective and specific deliverability against one or more malignant tumors in a patient likely to benefit clinically from its prescription [1]. In this article, a “theranostic radiopharmaceutical” is meant to define such an agent as possessing a radiochemical chelator or nonradioactive targeting ligand linked to (i) a diagnostic radionuclide for evaluating tumor uptake and any metastatic dissemination or (ii) a therapeutic radionuclide for treatment [1]. Here, we discuss a roadmap for a leveraged programmatic partnership for an academic theranostic cancer center. Factors influencing the operationalization of theranostic radiopharmaceuticals are also discussed using malignant neuroendocrine tumor and lutetium-177-(tetraazacyclododecanetetraacetic acid)-Tyr3-octreotate (DOTATATE) as illustrative examples.

## 2. Programmatic Collaboration

**Figure 1:**
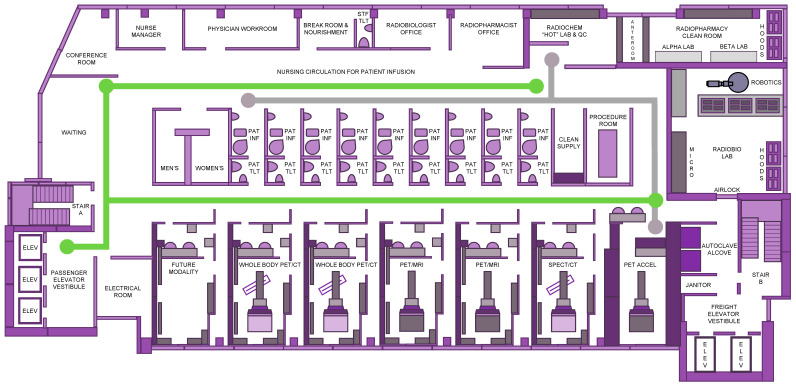
Theranostic cancer center floor plan. Illustrated are the elements of a theranostic cancer center floor plan with integrated patient and radiopharmaceutical spaces. The green path marks the pathways of patient travel through the center. Dedicated patient entry leads to a chic retail-like waiting room with check in kiosks and patient education materials. A small offset conference room allows for patient consultation or for morning staff-wide huddle. Informal nursing circulation with work kiosks flank nine patients appropriately shielded in radiopharmaceutical incubation/infusion rooms each with a dedicated bathroom. A patient minor procedure room rounds out the hallway. A nurse manager charged with an additional patient navigation role has prime office space, with physician workroom, staff break room, and radiobiologist office, as well as radiopharmacist office line the perimeter. A radiopharmacy engaged in the sterile and nonsterile handling of radioactive drugs has an anteroom, negative-pressure clean room with appropriate air quality, alpha and beta emission workspaces as well as lead-lined biologic safety cabinets. The gray path outlines radiopharmaceutical traffic as directed by the radiopharmacy. A robotic self-driven radiobiology laboratory, with a negative-pressure clean room and appropriate air quality, contains an immunohistochemical microscopy facility and complimentary high-performance liquid chromatography as well as liquid chromatography with tandem mass spectrometry. A biology-guided radiotherapy accelerator offers PET tracer-based localization and treatment of patient tumor. Two whole-body PET/CT, two PET/MRI and one SPECT/CT scanners provide diagnostic or dosimetry activity and evaluate radiopharmaceutical treatment effect.

Forward-thinking changes in the teamwork among medical oncology and radiation oncology as well as nuclear medicine teams demand a reexamination of the core concepts surrounding the patient experience, safety, and resource support for discovery-phase or development-phase radioactive agents. Thought leaders envision a centralized nonduplicative theranostic unit occupying a single cancer center floor rather than a siloed decentralized service (Figure 1). Because these drugs can be infused, injected, ingested, or inhaled such that they emit alpha-particles (i.e., helium nuclei emitted from the nuclei of radionuclides), beta-particles (i.e., electrons emitted from the nuclei of radionuclides), or conversion electrons (i.e., electrons emitted from electron shells of radionuclides) to cancer cells residing in tumors or circulating in the blood, care must be taken to limit patient undertakings with radioactive material transport for preclinical, diagnostic, or therapeutic activities (Figure 1). We suggest that single-floor theranostic center logistics and operations invite new opportunities for phase 0–I research programs, drawing upon a population of patients interested in aiding discovery of predictive pharmacodynamic biomarkers or in supporting development of radiopharmaceutical–agent combinations [5]. Such was our experience with the phase I study of triapine-[^177^Lu] lutetium-DOTATATE in a metastatic neuroendocrine patient trial that progressed from a phase I trial (NCT04234568) to a phase II randomized trial (NCT05724108).

When designing an academic theranostic center, care must be taken to discover any existing or the need to apply for a radioactive material license from the governing regulatory entity. Support for a radioactive material license draws from the existence of adequate infrastructure, adequate personnel (including trained authorized user physicians, nuclear technologists, nursing staff, a Radiation Safety Officer (RSO), a medical physicist), sufficient mechanisms of radiation protection, and standard operating procedures for the discharge management of radioactive patients and means of radioactive waste and sewage.

An advantage of programmatic collaboration would be within scope-of-practice medical monitoring of investigational radiopharmaceuticals used in early-phase or late-phase clinical trials [5]. The NCI, following the lead of the United States Food and Drug Administration (FDA) [6], instructed that no principal investigator lead a clinical investigation of a study intervention like a radiopharmaceutical drug until that individual has provided a completed, signed Statement of Investigator Form FDA 1572 [21 CFR 312.53(c)]. In its guidelines, the NCI stipulated that a principal investigator must supervise all aspects of the clinical investigation, follow protocol-only methodologies, inform subjects that investigational agents are being used for experimental purposes, ensure informed consent, report adverse events, and maintain adequate and accurate records. The guidelines intended that at least one radiation oncology and one nuclear medicine physician served as authorized users for the tasks of receipt, handling, preparation, dispensing and final disposition of the radiopharmaceutical as well as its medical monitoring during and after administration. Adherence with all Nuclear Regulatory Commission or agreement-state radiation material license regulations and rosters is expected. A single floor plan accommodates these suggestions (Figure 1).

Another key aspect envisioned for success in the programmatic effort would be the involvement of a nurse manager coordinating patient experience with patient navigation for diagnostic and therapeutic activities (Figure 1). There has been an emerging desire to reduce communication barriers to timely, efficient, and high-quality oncology cancer care; patient navigation has contributed to overcoming these obstacles [7,8]. We advocate that an oncology-certified nurse navigator might streamline diagnostic processes, coordinate patient support services and education, and arrange specialty consultation in medical oncology, nuclear medicine, and radiation oncology. It was thought that to succeed in the position, applicants would have a degree in nursing, an up-to-date unrestricted license for nursing practice, and a certificate in oncology nursing. Three years of practice experience in oncology nursing would be preferred. Duties of the nurse navigator would involve support for scheduling diagnostic imaging scans (e.g., whole-body positron emission tomography (PET)-computed tomography (CT), PET-magnetic resonance imaging (MRI), or single-photon emission computed tomography (SPECT/CT) scans) and diagnostic procedures (e.g., biomarker blood sampling and tissue biopsy). We felt that their patient-centered care role would include patient needs assessments, patient education, and psychosocial support, as well as providing care management through coordinated specialty oncology consultations via a specialty tumor board. A nurse manager in this role, with a centralized office integrated into the floor plan, has worked well before [9].

### 2.1. Patient Experience

With mounting out-of-pocket healthcare expenses, patients have become value-based healthcare consumers. To succeed, theranostic programs need to emulate a retail-like approach to radiopharmaceutical administration.

A desire to meet patient value-based health care has incentivized theranostic programs to consider merging high-end concierge services like cross-departmental digital check in kiosks in parallel with consumer-centric patient adverse event reporting technology for best supportive cancer treatment management. Radiopharmaceutical cancer treatment lends itself well to these tactical advantages because of drug-like pharmacology resulting from measurable pharmacokinetics and anticipatable organ toxicities. Seamless patient flow from chic waiting room lobbies to a sophisticated consultation conference room to private infusion, blood draw, or procedure rooms enables safe and time-efficient radioactive drug administration (Figure 1). 

We also advocate that individualizable technology like wearable wristband sensors or mobile phone applications could capture biometric data or patient-reported outcomes (PRO) prior to physician-patient appointments, all resulting in less time assessing cancer treatment symptom severity and trajectory and more time in management as a means of enriching the patient experience [10]. Take, for instance, a neuroendocrine cancer patient’s pretreatment grade 1 urinary frequency. In this case scenario, let us say that after treatment with the radiopharmaceutical [^177^Lu] lutetium-DOTATATE, their post-treatment urinary frequency rose to grade 2, required medication, and interfered with grocery shopping. A typical adverse event assessment would capture the objective change in urinary frequency due to a need for a medical prescription but would not necessarily identify the PRO adverse event as a disruption in an instrumental activity of daily living. A patient-centric experience tool like a mobile phone application offers better qualified data on how an individual participant lives during and after their radiopharmaceutical treatment. Such data could be banked at the theranostic center upon patient check-in and synchronization of their mobile phone application with the registration kiosk (Figure 1). Then, this could be made available readily to the caregiving nurse and physician. Such aesthetic advantages offer services and experiences that stand out, resonate, and delight healthcare customers and should be integrated into the theranostic center floor plan.

A practical aspect in the patient experience is the minimization of radiation exposure. Our roadmap for the patient experience (Figure 1, green pathway) involves shuttling patients away from potential sources of exposure. Consistent with other thought leaders building theranostic centers [11,12], the patient infusion rooms use appropriate shielding of radiopharmaceutical vials and syringes according to their emissions as well as shielding of designated storage and waste containers. This ranges from polymethyl metacrylate storage bins for vials or waste containers to lead pots or tungsten syringe shields to concrete waste bunkers and lead-lined treatment rooms [11], all according to local regulatory agency specifications for radioprotection. This also must consider the transport of radioactive material from the radiopharmacy to, from, and across the floor, with appropriate documentation, to secure the radioactive material from its source to its final use and waste (Figure 1, gray pathway). Radioactive agents used in the theranostics center must be stored in a safe, secure, environmentally appropriate place where only the licensee and appropriate designated staff have access [11,12]. Leveraged programmatic cooperation ensures these concepts are seamlessly integrated into the theranostic center floor plan.

### 2.2. Imaging Tools

A progressive approach to cancer detection proceeds stepwise from the evaluation of cellular-level changes by positron emission tomography to the determination of morphologic alterations by magnetic resonance imaging using a streamlined scanner (Figure 1).

Radiopharmaceuticals are decidedly precise, possess desired target in-residence, and have advantageous elimination properties that promote peak tumor-to-background delimitation. Diagnostic-therapeutic dyads aid in early cancer detection and treatment response by measuring biodistribution, drug-bound-to-target lifetime, dosimetry, and consequent biologic effect factors. Take, for example, the theranostic dyads of [^68^Ga] gallium-DOTATATE or [^64^Cu] copper-DOTATATE and [^177^Lu] lutetium-DOTATATE. Determining a radioactivity exposure window might be critical to the evaluation of radiopharmaceutical effect when used against neuroendocrine tumor(s)—a window surely open and closed by pharmacokinetic factors. Imaging by [^68^Ga] gallium- or [^64^Cu] copper-DOTATATE to ensure tumor positivity prior to [^177^Lu] lutetium-DOTATATE administration is sensible to make sure that the ^177^Lu payload “hits” targeted tumor(s). Afterward, dosimetry-based calculations on SPECT/CT scanners confirm irradiation dose delivered (Figure 1). It is intended eventually that [^68^Ga] gallium/[^64^Cu] copper-DOTATATE site intensity relative to normal tissue background will assess an individual patient’s tumor burden, target in-residence time, and tumor heterogeneity so that subsequent personalized radiopharmaceutical dose might be given for optimal tumor dose without undue harm to normal organs at risk [13,14]. In conventional drug discovery, judgements about priority therapeutic agent selection for development are weighed upon in vitro and in vivo data, which is problematic for radiopharmaceuticals because of the dangers of handling radioactivity in laboratories or clinics. As such, radiopharmaceutical safety and efficacy studies underperform, which may result in favorable agents not being developed fully. We contend that a theranostic center which integrates imaging elements with its radiopharmacy and radiobiology capacity to deliver essential pharmacokinetic and pharmacodynamic data informs decision-making for radiopharmaceutical discovery and development. 

Detailed protocols for molecular imaging and morphologic assessment are beyond the scope of this floor plan discussion. Because [^18^F] fluoro-2-deoxy-D-glucose (^18^F-FDG) radiotracers are typically employed (but realize that there are many emerging positron emitters available for molecular imaging), our ideal theranostic center has a footprint dominated with PET imagers (Figure 1) scheduled in 60 min patient time slots. A whole-body PET imaging protocol might entail multiple bed positions from the skull base through the thighs, lasting from as short as 5 min to as long as 45 min. A PET/MRI protocol could demand multiple morphologic sequences for the organ of interest, perhaps including a Dixon for attenuation correction, diffusion weighted imaging in free-breathing, axial free-breathing T1 gradient echo volumetric interpolated breath-hold examination (GRE VIBE), and free-breathing T2 turbo spin echo acquisition (TSE). PET/MRI acquisition might total 75 min. As clinically indicated for PET/MRI imaging, medications like furosemide for bladder lesions, gadolinium contrast like for neuroendocrine liver lesions, or changes in organ-specific protocols like a small field of view for pelvic tumors, augment these protocols. Our thoughts on these issues were developed from relevant guidelines [15,16,17,18,19,20].

### 2.3. Biology-Guided Radiotherapy

Postponing the cause-of-death trajectory of cancer by eliminating gross tumors near critical organs enables the benefits of systemic therapies whose effects are not immediate, like radiopharmaceuticals, biologics, or immune system modulators, to be realized.

An evolving management strategy for persistent, recurrent, or metastatic cancer involves irradiation of bulk tumors using stereotactic ablative radiotherapy (SABR) techniques. However, SABR of several disease foci with external beam radiotherapy platforms is onerous. This has the unintended consequence of narrowing research on the use of metastatic disease ablation among advanced-stage patients. Alternatively, biology-guided radiotherapy (BgRT) has emerged as an external beam radiotherapy platform merging (i) PET and CT scanners, successful modalities for visualizing cancer, with (ii) a linear accelerator-based radiation machine, an effective means of treating cancer [21,22]. A BgRT platform functions by setting patient position using kVCT imaging and then by detecting tumor location through PET tracer tumor emissions. Irradiation occurs from accelerator-generated radiation beamlets transpiring within sub-second latency (350–400 milliseconds) [21,22]. Sub-second tumor-accelerator feedback permits a very high radiation dose to the tumor but causes normal tissue dose falloff, even in moving targets. This also has the advantage of less radiation dose deposition in normal tissues than conventional radiotherapy. The key advantage of BgRT is the complete ablation of target based on direct biologic activity rather than indirect imaging; for this reason, a BgRT platform is included in our ideal floor plan (Figure 1).

In our opinion, for advanced-stage neuroendocrine malignancy patients, the BgRT approach fits into one of two therapeutic strategies—(i) a switch maintenance strategy, whereby after a first-line systemic chemotherapy regimen patients are switched to an BgRT and then radiopharmaceutical sequence until disease progression, or (ii) a continuation maintenance strategy, whereby one treatment of the first-line systemic chemotherapy regimen is continued past its standard duration alone or in a new combination until disease progression [23]. By locating a BgRT linear accelerator within the theranostic center and next to its partnering diagnostic scanners, there is the opportunity for quick adaptive radiotherapy plan modifications using a diverse radiotracer portfolio (Figure 1).

### 2.4. Radiobiology and Microscopy Laboratory

Radiopharmaceuticals entering early development-phase studies should declare a clear hypothesis supported by radiopharmacologic or radiobiologic basis complete with at least two cancer models each of in vitro and in vivo data.

Substantial investment in radiopharmaceuticals, alone or in combination, has occurred, and priority should be assigned to those that are linked to vetted molecular targets, have robust mechanistic validation, and are prospect for therapeutic success [1]. Nevertheless, the amount of data needed to predict therapeutic success remains unknown. We acknowledge that there is a changing use of two-dimensional cell culture (2D) and three-dimensional organoid coculture (3D) in vitro systems, and so, a theranostic center approach might be as follows.

We recommend that 3D in vitro cultures be studied first using radiopharmaceuticals at their clinically relevant concentrations alone or in combination with oncologic agents [1]. This means that the preclinical study involves two cancer cell lines of interest (i.e., two neuroendocrine cancer cell lines for a neuroendocrine cancer trial), and data should not be extrapolated from unrelated cell lines [1]. Patient tumor-derived organoid models might best estimate cellular, oxygenation, or spatiotemporal factors relevant for radiopharmaceutical radiosensitivity [1]. In vitro metabolic or clonogenic survival techniques are a valid measure of cytotoxic effect size; however, we encourage any metabolic cytotoxic effect should be affirmed by a clonogenic survival assay, as it assays three-to-four logs of cell kill and not just metabolic growth arrest [1]. A radiobiology laboratory near the radiopharmacy affords scientists access to short-lived radiopharmaceuticals on demand, reduces radionuclide transport to as low as reasonably acceptable levels, and provides the practical step to narrow down effective dosages and schedules that will undergo investigational new drug (IND)-enabling toxicology and eventual clinical testing (Figure 1). However, the radiobiology laboratory is a separate entity distinct from clinical areas and possesses an air handling system that prevents cross-contamination. 

The radiobiology laboratory would engage advanced robotic systems to (i) physically connect the stages of radiopharmaceutical synthesis, characterization, and performance evaluation in the radiopharmacy and radiochemistry laboratory, as well as (ii) reduce the time gap between performing an experiment and deciding the next step in preclinical development. Self-driving laboratories integrate machine-learning, lab automation, and robotics and engage non-oncology experts into the leveraged radiopharmaceutical program [24]. Such robotic systems might explore relations to radioactive drugs used in the clinic and biologic insights from disease responses such as the through use of high-performance liquid chromatography or liquid chromatography with tandem mass spectrometry. In the modern theranostics center, a self-driving laboratory would avail the radiopharmaceutical team substantial time to focus upon new therapeutic questions and treatments rather than spending time on time-consuming repetitive laboratory tasks [24]. Intelligent experimental plans and autonomous experiments along the target identification to in vitro to in vivo testing spectrum allows big picture bench-to-bedside therapy integration in the modern theranostic center [24,25,26].

### 2.5. Radiopharmacy and Radiochemistry

Radiopharmaceuticals for clinical use should be dispensed by a radiopharmacy (i.e., a specialty area of pharmacy practice) engaged in the preparation of radioactive drugs to improve and to promote health through the safe and the effective use of these agents to diagnose and treat specific disease states. Our theranostic center’s footprint initially anticipates the delivery of radiopharmaceuticals from an off-site medical cyclotron but also permits growth for a medical cyclotron to be located in close proximity (Figure 1).

The goals and objectives of a radiopharmacy and radiochemistry laboratory are changing to include further evaluation of radiopharmaceuticals such as molecular target radiobiology in addition to dose-adverse event profiles of investigational agents. Because of these changes in focus, the overarching vision for the radiopharmacy and radiochemistry laboratory should gauge the most efficient way to receive, prepare, and dispense radiopharmaceuticals at its onset but then should evolve into a dynamic pharmacy for new molecular entity creation. Clinical approach to the pharmacy should contribute to phased study of conventional and investigational agents, with all intending dose and schedule determination, patient safety, and limited exposure to ineffectual doses of therapeutic radiopharmaceuticals [27,28,29,30].

The radiopharmacy and radiochemistry laboratory should have sufficient storage for decay of radiopharmaceuticals segregated by the expected time required for their decay. There should be sufficient storage for potentially contaminated items like patient clothing. Aqueous radioactive agent disposal into public sewage may be permissible with highly diluted wastewater, but regulatory authorities might request the environmental and radiologic impact of such behavior.

The radiopharmacist at the leading edge of therapeutic radioactive drug development should have a working knowledge of pharmaceutical sciences, including microbiology, chemistry, physiology, and pharmacology, along with basic radiation physics to provide essential background for the support of nursing, physician, and scientist endeavor. This intends that their practical skills in aseptic manipulation, in the safe handling of radioactive drug products, and in clinical management are integrated formally into the theranostic program. It is also anticipated that their knowledge of analytical techniques, including chromatography, gel filtration, and electrophoresis, useful in relation to quality control, are complementary to radiobiologist activities. 

The radiopharmacist should be a team member aiding in the release of patients. After diagnostic procedures, there are no extensive measures for the release of patients since the physical and effective half-lives of the radiotracers involved are only a few hours. The release situations are different for patients discharged after therapeutic administrations, as the higher activity levels demand alternative dose limits. A dose limit of one (1) mSv per year for the public and a dose constraint of five (5) mSv per episode for caregivers (a family member or paid helper who regularly looks after a child or a sick, elderly, or disabled person) have been proposed as acceptable limits [11,12]. The issues around the release of patients after treatment are well summarized elsewhere [11,12].

## 3. Discussion

The entrepreneurial success of a theranostic center is contingent upon the degree of partnership among several oncologic practices [1]. Most radiopharmaceutical clinical care integrates nursing and radiation safety personnel in all aspects of a leveraged program. The targeted agents given to patients for which all effects are not fully defined, despite intense prospective study, necessitates practical nursing and radiation safety effort during patient care. As such, patient with a variety of different malignancies that are either refractory to therapy or widely disseminated are often recruited to radiopharmaceutical therapies. These factors raise the importance of nursing and radiation safety in the practice of radioactive therapy.

Radiopharmaceuticals that can be given with precise selectivity for a patient population whose tumors are driven by the target of interest are rare. This situation is not surprising given that advanced-stage cancers are heterogeneous in molecular profiles such that targeting a single or a few relevant radiobiologic targets might be ineffective. Even for a radioactive drug developed along the phase 0–I to phase III trajectory, the personalization of drug dose is uncommon [27]. Dosimetry of the radioactive agent informs efforts to personalize the dose and is a key factor in future clinical benefit [27]. We argue that diagnostic imaging as well as radiopharmaceutical treatment are now embedded within community oncology workflows; facilities that provide discovery-phase and development-phase activities together offer the greatest clinical return on investment and expansion of a cutting-edge research portfolio. It does not surprise us that the currently most successful theranostic centers are embedded in strong cancer centers focusing on neuroendocrine tumors and with expansive potential to other solid tumors. As theranostic agent indications expand, a close collaboration with all clinical stakeholders involved in the management of cancer patients remains very important. 

## 4. Conclusions

Radiation oncologists, radiopharmacists, radiobiologists, nuclear medicine physicians, and medical oncologists participate together in the resurgence of therapeutic radiopharmaceuticals for the curative or the palliative management of cancer. In our view, the radioactive agents are attractive in the era of therapeutic personalized medicine because of the vast number of exploitable radiobiologic antigen targets available against cancer. A modern retail-like experience for patients and their caregiver’s workflow adds consumer-centric value to a theranostic program. The clinical utilization of PET/CT and PET/MRI scanners are important elements to evaluate the clinical performance of radiopharmaceuticals and need further study in this aspect. Integration of BgRT biotechnology for the treatment of bulky advanced-stage disease opens new pathways of therapy and clinical investigation. A comprehensive radiopharmacy and radiobiology laboratory unit allows for the on-site delivery of radiopharmaceuticals, drug discovery, and trial development. Overall, leveraged programmatic collaboration drives brand-new treatments early and often to patients. 

## Data Availability

All data generated or analyzed during this study are included in this published article.
